# Lupus nephritis and its association with subclinical myocardial alterations in systemic lupus erythematosus assessed by cardiovascular magnetic resonance

**DOI:** 10.3389/fimmu.2026.1749478

**Published:** 2026-02-06

**Authors:** Zhi Yang, Ling-li Wang, Tian-yue Zhang, Miao Wen, Hao Zou, Yong Zhu, Xue Meng, Liang-chao Gao, Bing Fu, Shu-yue Pan

**Affiliations:** 1 Department of Radiology, Chengdu Fifth People’s Hospital, Chengdu, China; 2 Department of Rheumatology and Immunology, Chengdu Fifth People’s Hospital, Chengdu, China

**Keywords:** cardiovascular magnetic resonance imaging, late gadolinium enhancement, strains, systemic lupus erythematosus, T1 mapping, T2 mapping

## Abstract

**Background:**

Lupus nephritis (LN) is the most common and severe complication in patients with systemic lupus erythematosus (SLE) and is associated with cardiac disease. The purpose of this study was to assess the cardiac phenotype of SLE patients with LN using cardiovascular magnetic resonance (CMR), and to investigate whether comorbid LN is associated with left ventricular (LV) remodeling.

**Methods:**

Clinical assessment and CMR were performed in 66 SLE patients without LN, 36 SLE patients with LN, and 20 age- and sex-matched healthy subjects.

**Results:**

SLE patients with LN had a more impaired global longitudinal strain (-12.37 ± 5.15% vs. -14.40 ± 2.80% vs. -14.92 ± 3.04%; P = 0.045) than SLE patients without LN and control group. Moreover, native T1 (1330 ± 54 vs. 1286 ± 81 vs. 1256 ± 41; P<0.001), extracellular volume (ECV) (30.53 ± 4.57% vs. 28.34 ± 3.59% vs. 26.20 ± 3.03; P<0.001), and native T2 (43.69 ± 4.32 vs. 41.98 ± 3.66 vs. 39.60 ± 2.94; P<0.001) were higher in SLE patients with LN, intermediate in SLE patients without LN and lowest in control group. However, LV-LGE did not differ significantly between the SLE patients with or without LN (P > 0.05). In multivariable linear regression, LN status was associated with higher native T1 (β=0.244, P<0.05) and ECV values (β=0.224, P<0.05).

**Conclusions:**

SLE patients with LN showed more pronounced subclinical myocardial abnormalities on CMR. LN was an independent risk factor for cardiac impairment in patients with SLE.

## Introduction

Systemic lupus erythematosus (SLE) is a systemic autoimmune disease with multiple organ involvement, especially in females (1). Importantly, SLE has a strong tendency to cause lupus nephritis (LN), which is the most common and severe complication (2). In addition, SLE patients with LN have a markedly higher risk of cardiovascular disease than those without LN, including myocardial infarction, heart failure and cardiovascular mortality (3). Indeed, the pathogenic mechanism underlying the elevated risk of cardiovascular disease in SLE patients with LN is still not fully elucidated. Moreover, the leading cause of mortality in patients with SLE and LN is cardiovascular disease (4). Therefore, early identification of cardiovascular disease and close monitoring of myocardial involvement are key to improving outcomes, especially for patients with SLE and LN.

To date, cardiovascular magnetic resonance (CMR) imaging is considered a reliable non-invasive modality for the early screening and evaluation of myocardial involvement in patients with SLE (5). Previous studies have confirmed that CMR can detect myocardial inflammation, fibrosis, and edema using late gadolinium enhancement (LGE), and T1 and T2 mapping sequences during the subclinical period of myocardial injury (6–9). In particular, strain analysis based on CMR provides additional diagnostic value beyond left ventricular ejection fraction (LVEF) and LGE in patients with SLE (7).

In clinical practice, understanding the associations between LN and cardiovascular disease is important to clarify the mechanisms underlying the pathogenesis of major adverse cardiovascular events in patients with SLE and LN. However, the use of CMR to assess myocardial involvement in SLE patients with LN remains limited. Moreover importantly, it remains unclear whether the presence of LN is independently associated with myocardial function in patients with SLE. Therefore, the objective of this study was to evaluate myocardial status in SLE patients with LN using CMR, and to investigate whether LN is an independent contributing factor to myocardial involvement.

## Methods

### Study population

This was a retrospective study conducted at the Chengdu Fifth People’s Hospital between January 2021 and May 2024. We retrospectively identified consecutive SLE patients who underwent CMR during the study period and met the eligibility criteria. SLE was classified according to the 2019 European League Against Rheumatism/American College of Rheumatology classification criteria (10). All participants were aged ≥18 years. Exclusion criteria included pregnancy and pre-existing cardiac disease prior to CMR (coronary artery disease/myocardial infarction, cardiomyopathy/heart failure, moderate-to-severe valvular disease, congenital heart disease, cardiac sarcoidosis, cardiac amyloidosis, or other established structural heart disease)(**Supplementary Figure S1**). SLE patients with hypertension were defined using the following criteria: resting systolic blood pressure >140 mm Hg or diastolic blood pressure >90 mm Hg or use of antihypertensive medication. In addition, LN was defined as either (1) biopsy-proven LN, or (2) clinically suspected renal involvement with proteinuria meeting the renal criterion (24-hour urinary protein ≥0.5 g and/or urine protein-to-creatinine ratio ≥0.5), supported by documentation of active urinary sediment (e.g., hematuria and/or red blood cell casts) and/or serologic activity consistent with SLE (e.g., low C3/C4 and anti-dsDNA positivity/elevation when available), with alternative causes of renal impairment excluded based on clinical record review (10–12). Consequently, the SLE patients were divided into two subgroups: the SLE patients with LN group and the SLE patients without LN group. For comparison, a control group of healthy subjects was enrolled with a similar age and sex distribution to the SLE patients. All of the healthy subjects in the control group underwent CMR examinations and had normal clinical and laboratory findings and no history of cardiovascular or metabolic disease. Clinical characteristics (including age, sex, and cardiovascular risk factors [e.g., hypertension]) as well as laboratory data and medication information were retrieved from the electronic medical record system. Laboratory indices were obtained from the closest available assessment to the CMR examination (within 7 days). Our study was approved by the Biomedical Research Ethics Committees of our hospital, and written informed consent was obtained from all patients.

### CMR image acquisition

All individuals underwent CMR using 3.0T MRI system (Vida; Siemens Medical Solutions, Germany) with 32-channel body coil. ECG-gated, breath-hold were used to obtained cine images. Cine, T2 mapping, T1 mapping and LGE images were acquired in standard long-axis (2-chamber and 4-chamber) and contiguous short-axis views covering the entire left ventricle (LV). Cine images were acquired using the balanced steady state free precession (SSFP) sequence with the following parameters: repetition time (TR): 39.12 ms; echo time (TE): 1.43 ms; flip angle (FA): 80°; field of view (FOV): 420 mm; matrix: 256×199; phase: 25. Cine images such as short-axis stack covering the entire LV (8 mm slices with 2 mm gap).

Native and post-T1 mapping was performed using a modified Look-Locker Inversion Recovery (MOLLI) technique with the following parameters: TR: 257.3 ms; TE: 0.95 ms; FA: 35°; FOV: 420 mm; matrix: 256×144; Bandwidth: 1085 Hz/Px; Echo Spacing: 2.24 ms, in three short-axis slices (basal, mid-ventricular, and apical levels). Post-contrast T1 mapping was acquired 10–15 minutes after intravenous administration of gadolinium diethylenetriamine penta-acetic acid (Gd-DTPA; MultiHance; Bracco) at a dose of 0.2 mmol/kg, according to standard CMR recommendations. T2 map using a T2-prepared sequence with the following parameters: TR: 224.8 ms; TE: 1.22 ms; FA: 12°; FOV: 420 mm; matrix: 192×116; Bandwidth: 1184 Hz/Px; Echo Spacing: 2.94 ms and matched to the native T1 map. LGE images were acquired 10–15 minutes after Gd-DTPA injection using the phase-sensitive inversion recovery (PSIR) sequence with the following parameters: TR: 740 ms; TE: 1.06 ms; FA: 40°; FOV: 420 mm; matrix: 256×144.

### CMR analysis

All CMR images were analyzed using CVI42 software (CMR42, v. 5.15.4, Circle Cardiovascular Imaging, Calgary, Canada) by a radiologist with 5 years of CMR experience who was blinded to the clinical information.

The short-axis cine stack was loaded into the functional SAX module and the endocardial and epicardial borders were manually delineated at the LV end-systolic and end-diastolic phases. LV and right ventricular (RV) functional parameters, including left ventricular ejection fraction (LVEF), left ventricular end-diastolic volume (LVEDV), left ventricular end-systolic volume (LVESV), left ventricular stroke volume (LVSV), left ventricular mass, right ventricular ejection fraction (RVEF), right ventricular end-diastolic volume (RVEDV), right ventricular end-systolic volume (RVESV), right ventricular stroke volume (RVSV) and LV mass, were automatically calculated by the software. The papillary muscles were included in the LV volume.

The LV global strain parameters including global radial strain (GRS), global circumferential strain (GCS) and global longitudinal strain (GLS), were calculated by the feature-tracking module using short-axis, 2-chamber, and 4-chamber long-axis cine images. Native T1 and extracellular volume (ECV) were obtained on native and post-T1 mapping using tissue T1 mapping module. T2 values were calculated by manually tracing the endocardial and epicardial borders using the tissue T2 mapping module, and the global T2 values were obtained.

The initial assessment of LGE images was conducted through a visual examination. In the event of LGE being present, its extent was quantified using the tissue signal intensity module with a five-standard-deviation (5-SD) threshold above remote normal myocardium, and expressed as a percentage of LV mass.

### Statistical analysis

Statistical analyses were performed using GraphPad Prism 7 (Version 7.00, GraphPad Software, Inc.) and SPSS (Version 23.0, Released 2015, IBM Corp). The normality of the data was tested using the D’Agostino & Pearson normality test, and the results were presented as either the mean with SD or the median with interquartile range, depending on the characteristics of the data set. Categorical data are presented as numerical values and as percentages. Parameters among the controls, SLE without LN group and SLE with LN group were compared by one-way analysis of variance (ANOVA) for normally distributed variables or the Kruskal–Wallis test for non-normally distributed variables. When the overall test was significant, *post hoc* pairwise comparisons were performed using Tukey’s test (after ANOVA) or Dunn’s test (after Kruskal–Wallis). Categorical variables were compared using the χ² test or Fisher’s exact test, as appropriate. Univariable and multivariable linear regression were employed to ascertain the independently associated factors of the LV LGE extent, ECV, T1 and T2 values. Univariable and multivariable linear regression analyses were performed among SLE patients to identify factors associated with LV LGE extent, native T1, ECV, and native T2. For each outcome, candidate covariates were entered into the multivariable model if they showed a univariable association at P < 0.10 and/or were deemed clinically relevant, including hypertension, sex, age, disease duration, LVEF, LV mass index, and LN status, with attention to collinearity. Model fit was summarized using R². P value less than 0.05 was considered statistically significant.

## Results

### Studied population

Overall, 122 subjects (102 patients with SLE and 20 controls) who met the inclusion and exclusion criteria were included. Among the patients with SLE, 36 (35%) were classified as having LN, and 66 (65%) were classified as SLE without LN. Among the 36 patients in the LN group, 16 (44.4%) had biopsy-proven LN, while the remaining patients met the clinical definition based on proteinuria ≥0.5 g/24 h.

### Patient characteristics

The characteristics of the study participants are shown in **Table 1**. Age, sex, disease duration, systolic blood pressure and diastolic blood pressure did not differ significantly between SLE patients with and without LN (P > 0.05). However, hypertension was more common in SLE patients with LN than in those without LN (P < 0.05). As expected, immune and renal parameters such as neutrophils, erythrocyte sedimentation rate, creatinine and urea nitrogen levels were higher in the LN group than in the non-LN group (P < 0.05).

**Table 1:** Baseline characteristics in SLE with or without LN.

Variables	Control (n=20)	SLE without LN (n=66)	SLE with LN (n=36)	P value*
Age, years	45.95 ± 15.00	43.76 ± 13.99	38.36 ± 13.77	0.089
Female, n (%)	16(80.00)	61(92.42)	32(88.89)	0.286
Disease duration, years	-	1.00(0.17,6.00)	3.00(0.16,10.00)	0.213
Hypertension, n (%)	–	8(12.12)	16(44.44)	**<0.001**
Systolic blood pressure (mmHg)	-	123.6 ± 14.11	125.8 ± 17.87	0.502
Diastolic blood pressure (mmHg)	–	80.43 ± 12.72	80.89 ± 15.08	0.871
Clinical				
Laboratory results				
White cell count (×10^9^/L)	-	4.93(3.27, 7.17)	4.83(3.55, 7.30)	0.844
Neutrophils (%)	–	68.55(61.50, 79.63)	76.15(68.50, 82.85)	**0.028**
Hemoglobin (g/dl)	-	114.0 ± 22.65	103.6 ± 20.36	**0.004**
Hematocrit (%)	–	36.81 ± 8.93	31.64 ± 5.89	**<0.001**
CRP (mg/L)	-	3.00(1.02, 13.85)	6.30(2.00, 18.40)	0.150
ESR (mm/1^st^ hour)	–	33.00(12.00, 62.25)	41.00(20.75, 104.8)	**0.030**
Urinary protein positive, n (%)	-	11(18.64)	29(80.56)	**<0.001**
Creatinine (mg/mL)	–	54.95 ± 13.86	83.66 ± 33.19	**<0.001**
Urea nitrogen (pg/mL)	-	5.57(4.17, 7.46)	6.59(5.04, 9.98)	**0.033**
Serum albumin (g/L)	–	38.9(35.6, 40.5)	28.8(25.2, 36.1)	**<0.001**
Glutamic-pyruvic transaminase (U/L)	-	21.00(15.00, 38.00)	20.00(12.25, 36.00)	0.355
Glutamic oxaloacetic transaminase (U/L)	–	25.00(19.50, 33.50)	25.00(19.00, 42.75)	0.706
Blood glucose (mg/dL)	-	5.08(4.56, 6.00)	4.72(4.08, 5.49)	**0.043**
Total cholesterol (mmol/L)	–	4.22 ± 0.95	4.55 ± 1.77	0.894
Triglyceride (mmol/L)	-	1.40(1.06, 2.06)	1.90(1.46, 2.74)	**0.016**
HDL-cholesterol (mmol/L)	–	1.22 ± 0.44	1.02 ± 0.35	**0.026**
Blood calcium (mmol/L)	-	2.17 ± 0.11	1.99 ± 0.19	**<0.001**
Blood phosphorus (mmol/L)	–	1.15 ± 0.22	1.18 ± 0.25	0.308
Blood potassium (mmol/L)	-	3.71 ± 0.35	3.84 ± 0.62	0.761
Blood sodium (mmol/L)	–	141.9(140.5, 144.2)	140.2(137.3, 144.1)	**0.041**
Serum C3 (mg/L)	-	0.74 ± 0.28	0.63 ± 0.31	0.111
Serum C4 (mg/L)	–	0.13 ± 0.08	0.09 ± 0.07	**0.045**
ANA positive, n (%)	-	63(95.45)	33(97.06)	>0.999
Anti-dsDNA positive, n (%)	–	32(52.46)	19(59.38)	0.661
ANCA positive, n (%)	-	24(40.00)	13(39.39)	>0.999
ACA-IgG positive (U/L)	–	11.26(2.58, 15.89)	7.54(4.18, 83.71)	0.709
Creatine Kinase (U/L)	-	38.00(24.75, 67.00)	49.50(29.75, 91.50)	0.194
CK-MB (U/L)	–	10.00(8.00, 13.00)	10.00(9.00, 15.00)	0.459
Lactate dehydrogenase (U/L)	-	207.0(170.0, 259.0)	252.0(199.0, 351.0)	**0.011**
Medicine, n (%)				
Prednisone	–	50(50.00)	32(88.89)	**<0.001**
Hydroxychloroquine	-	54(81.82)	34(94.44)	0.129
Methotrexate	–	2(3.03)	2(5.56)	0.612
Tacrolimus	-	0(0)	1(2.78)	0.352
Rebamipide	–	24(36.36)	15(41.67)	0.671

Values are mean ± SD, number (%), or median (25th-75th percentile). Numbers in boldface indicate P values <0.05.

*P value across the 3 groups.

SLE, systemic lupus erythematosus; LN, lupus nephritis; CRP, c-reactive protein; ESR, erythrocyte sedimentation; HDL, high-density lipoprotein; ANA, antinuclear antibody; ANCA, Antineutrophil Cytoplasmic Autoantibodies; ACA-IgG, anti cardiolipin antibody-immunoglobulin G; CK-MB, creatine kinase-MB.

In terms of lipid profiles, triglyceride levels were higher and high-density lipoprotein cholesterol levels were lower in SLE patients with LN than in those without LN (P < 0.05). In addition, proteinuria was present in 29 LN patients (81%) and was significantly more frequent in the LN groups (P < 0.001). In the LN group, hemoglobin, hematocrit, serum albumin, blood calcium, and blood sodium levels were significantly lower than those in the non-LN group (P < 0.05).

Moreover, C4 levels were significantly lower in SLE patients with LN than in those without LN (P < 0.05). Cardiac enzymes, including creatine kinase and creatine kinase-MB, were within the normal range in both SLE groups and did not differ significantly (P > 0.05). However, lactate dehydrogenase levels were higher in the LN group than in the non-LN group (P < 0.05). Additionally, SLE patients with LN were more likely to receive prednisone medication than SLE patients without LN (P < 0.05).

### Cardiac function and LV strains

The cardiac MRI findings for the included subjects are shown in **Table 2**. The SLE patients with LN were comparable in LVEDV, LVESV, LVSV, LVEF, LV mass, LVMI, RVEDV, RVESV, RVSV, RVCO, and RVEF, with no significant difference among the three groups (P > 0.05). LVCO was higher in SLE patients with LN than in those without LN, although values remained within the normal range (P < 0.05).

**Table 2:** Baseline cardiac MRI characteristics in SLE with or without LN.

Variables	Control (n=20)	SLE without LN (n=66)	SLE with LN (n=36)	P value*
CMR				
HR,	71.70 ± 12.76	74.03 ± 13.44	74.05 ± 11.14	0.823
Pericardial effusions	0(0)	9(13.64)	10(27.78)	0.079
LV morphology & function				
LVEDV, ml	102.0 ± 22.9	104.8 ± 30.4	116.7 ± 25.5	0.074
LVESV, ml	38.28 ± 12.17	37.54 ± 17.16	43.15 ± 15.40	0.091
LVSV, ml	63.50 ± 13.97	67.19 ± 18.33	73.59 ± 18.92	0.094
LVCO, ml	4.51 ± 1.20	4.94 ± 1.51	5.31 ± 1.11‡	**0.044**
LVEF, %	62.55 ± 6.76	64.34 ± 7.38	63.24 ± 9.63	0.480
LV mass, g	63.62(52.15, 76.39)	64.44(55.52, 77.48)	71.28(62.98, 84.05)	0.073
LVMI, g/ml	0.63(0.56, 0.71)	0.62(0.55, 0.68)	0.63(0.57, 0.66)	0.985
LV strain				
GRS, %	40.01 ± 12.65	35.68 ± 11.17	34.99 ± 9.11	0.367
GCS, %	-18.88 ± 2.56	-19.47 ± 2.83	-19.27 ± 3.04	0.506
GLS, %	-14.92 ± 3.04	-14.40 ± 2.80	-12.37 ± 5.15	**0.045**
RV morphology & function				
RVEDV, ml	104.1 ± 26.46	113.5 ± 27.80	115.4 ± 28.10	0.330
RVESV, ml	48.52 ± 13.65	51.31 ± 18.95	51.96 ± 19.62	0.934
RVSV, ml	55.58 ± 15.27	62.20 ± 15.43	63.45 ± 20.18	0.238
RVCO,	3.90 ± 1.20	4.55 ± 1.21	4.59 ± 1.43	0.128
RVEF, %	50.53 ± 13.97	55.49 ± 9.16	55.06 ± 12.15	0.575
LV myocardial LGE				
LGE present, n (%)	–	19(28.79)	11(30.56))	0.851
LGE extent, %	-	0(0, 1.48)	0(0, 2.45)	0.535

Values are mean ± SD, number (%), or median (25th-75th percentile). Numbers in boldface indicate P values <0.05.

*P values are for overall comparisons across the three groups.

†P < 0.05 for pairwise comparisons versus the control group.

‡ P < 0.05 for pairwise comparisons between the SLE with LN group and the SLE without LN group.

SLE, systemic lupus erythematosus; LN, lupus nephritis; MRI, magnetic resonance imaging; CMR, cardiac magnetic resonance; HR, heart rate; LV, left ventricular; LVEDV, left ventricular end-diastolic volume; LVESV, left ventricular end-systolic volume; LVSV, left ventricular stroke volume; LVCO, left ventricular cardiac output; LVEF, left ventricular ejection fraction; GRS, global radial strain; GCS, global circumferential strain; GLS, global longitudinal strain; RV, right ventricular; RVEDV, right ventricular end-diastolic volume; RVESV, right ventricular end-systolic volume; RVSV, Right ventricular stroke volume; RVCO, right ventricular cardiac output; RVEF, right ventricular ejection fraction; LGE, late gadolinium enhancement.

**Figure 1:**
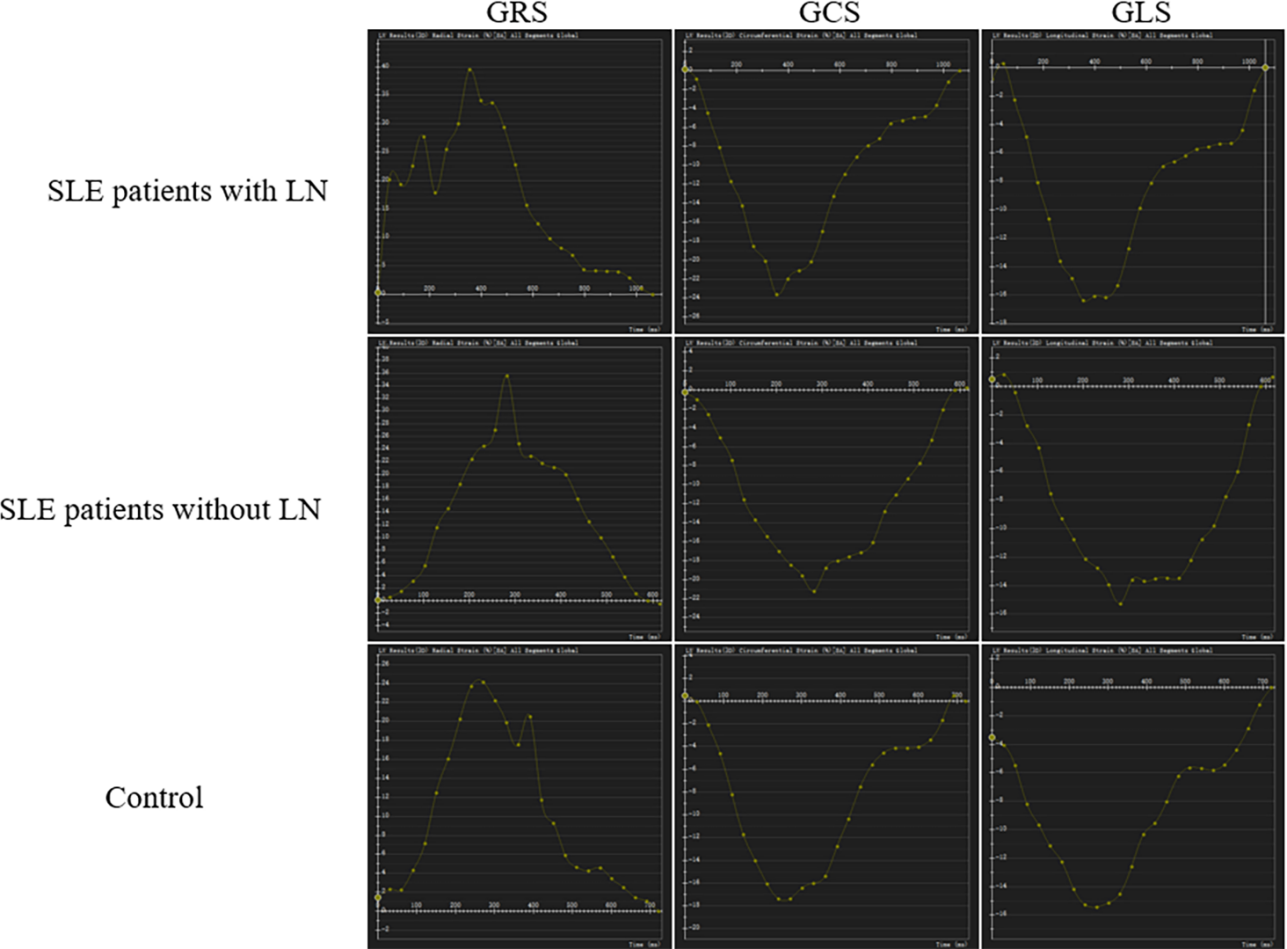
Representative three-dimensional global longitudinal strain in the left ventricle (LV) of systemic lupus erythematosus (SLE) patients with or without lupus nephritis (LN) and control. GRS: global radial strain; GCS: global circumferential strain; GLS: global longitudinal strain.

Moreover, we compared LV strain parameters among controls, SLE without LN, and SLE with LN. GRS and GCS were comparable between control group, SLE without LN, and SLE with LN groups (P > 0.05). In contrast, GLS was least negative in SLE patients with LN, more negative in SLE patients without LN, and most negative in controls (P < 0.05) (**Figure 1**).

### Diffuse myocardial involvement

In the present study, 30 SLE patients (29.41%) had LGE. Among the SLE patients with LN, 11 patients (30.56%) were LGE positive. However, the presence of LGE was comparable between SLE patients with LN and those without LN (P > 0.05). In addition, the extent of LGE did not differ significantly between the SLE patients with LN and those without LN (P > 0.05).

**Figure 2:**
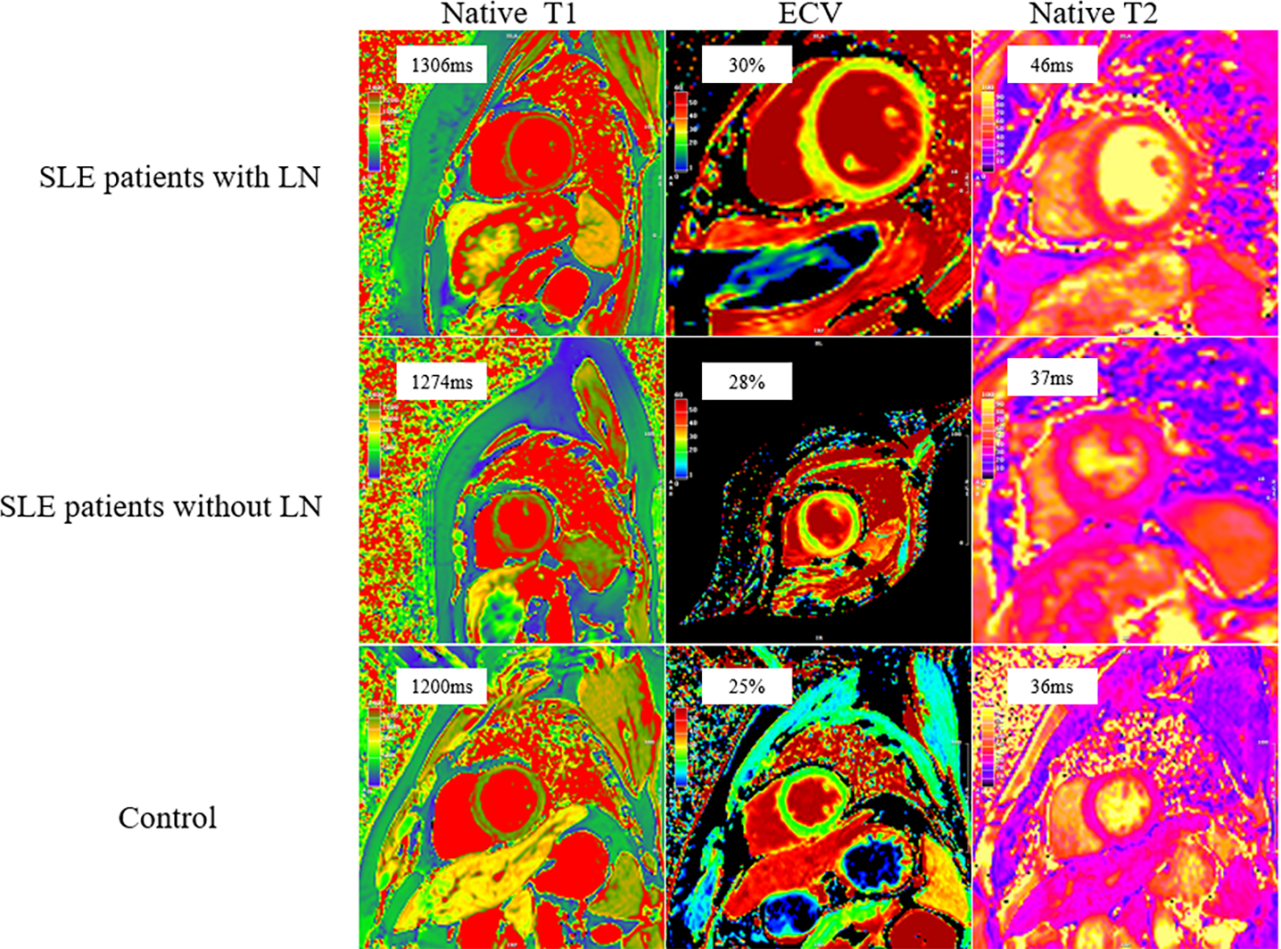
Distribution of native T1, T2 values and extracellular volume (ECV) of systemic lupus erythematosus (SLE) patients with or without lupus nephritis (LN) and controls. Note that global native T1, ECV, and native T2 were higher in systemic lupus erythematosus (SLE) patients with lupus nephritis (LN), intermediate in SLE patients without LN and lowest in control.

Notably, native T1, ECV, and native T2 values were highest in the SLE with LN group, intermediate in the SLE without LN group, and lowest in the control group (P < 0.05) (**Figure 2**). The multivariable linear regression analysis showed that the presence of LN was independently associated with increased native T1 (β = 0.244, P < 0.05) and ECV values (β = 0.224, P < 0.05). Furthermore, the presence of hypertension was also associated with increased LGE extent (β = 0.265, P < 0.05) (**Table 3**). Overall, mapping values were higher in the LN group, and similar directions were observed in exploratory analyses stratified by hypertension status (**Table 4**).

**Table 3:** Linear regression analyses of LV LGE extent and CMR mapping parameters in SLE patients.

Variables	LGE extent		Native T1		ECV		Native T2	
	Univariable β	Multivariable β, R2 = 0.147	Univariable β	Multivariable β, R2 = 0.162	Univariable β	Multivariable β, R2 = 0.178	Univariable β	Multivariable β, R2 = 0.110
Sex (Female)	-0.237*	-0.170	0.136		0.047		0.047	
Age	-0.015		-0.336*	-0.252*	-0.180**	-0.139	-0.291*	-0.228*
Disease duration >5 years	-0.122		-0.103		-0.130		0.035	
Hypertension	0.337*	0.265*	0.013		0.006		0.028	
LVEF <55%	0.060		0.065		-0.071		-0.021	
LVMI	-0.067		-0.188**	-0.110	-0.119		-0.281*	-0.217*
LN	0.195**	0.089	0.279*	0.244*	0.249*	0.224*	0.207*	0.147

Values are mean ± SD, number (%), or median (25th-75th percentile).

Numbers in boldface and with * indicate P values <0.05. Numbers with ** indicate P values <0.10.

LV, left ventricular; LGE, late gadolinium enhancement; SLE, systemic lupus erythematosus; LVESV, left ventricular end systolic volume; RVEF, right ventricular ejection fraction; LN, lupus nephritis.

**Table 4:** Impact of hypertension on LV myocardium in patients with SLE.

Variables	Healthy controls (n=20)	SLE patients with hypertension			SLE patients without hypertension		
		Without LN (n=8)	With LN (n=16)	P*	Without LN (n=58)	With LN (n=20)	P*
LV strain							
GRS	40.01 ± 12.65	34.93 ± 11.75	35.93 ± 9.38	0.662	35.79 ± 11.19	34.11 ± 9.05	0.326
GCS	-18.88 ± 2.56	-20.53 ± 3.07	-19.10 ± 3.72	0.441	-19.32 ± 2.79	-19.34 ± 2.33	0.776
GLS	-14.92 ± 3.04	-14.10 ± 3.47	-12.89 ± 3.02	0.151	-14.44 ± 2.72	-11.88 ± 6.63	0.154
LV LGE							
LGE present	0(0)	2(25.00)	7(43.75)	0.371	17(29.31)	4(20.00)	0.418
LGE extent	–	1.35 ± 3.25	4.72 ± 6.89	0.266	0.56 ± 1.25	0.95 ± 1.88	0.483
LV tissue characterization							
Native T1	1256 ± 41	1259 ± 64	1325 ± 48†‡	**<0.001**	1290 ± 83	1334 ± 60†‡	**0.004**
ECV	26.20 ± 3.03	27.50 ± 2.87	30.06 ± 3.76†	**0.004**	28.55 ± 3.64†	30.90 ± 5.19†	**<0.001**
Native T2	39.60 ± 2.94	41.88 ± 3.09	43.25 ± 3.51†	**0.005**	42.00 ± 3.76†	44.05 ± 4.94†‡	**0.002**

Values are mean ± SD, number (%), or median (25th-75th percentile). Numbers in boldface indicate P values <0.05.

*P values are for overall comparisons across the three groups.

†P < 0.05 for pairwise comparisons versus the control group.

‡ P < 0.05 for pairwise comparisons between the SLE with LN group and the SLE without LN group.

LV, left ventricular; SLE, systemic lupus erythematosus; LN, lupus nephritis; GRS, global radial strain; GCS, global circumferential strain; GLS, global longitudinal strain; LGE, late gadolinium enhancement; ECV, extracellular volume.

### Impact of LGE on the myocardium

In the SLE patients with LGE group, although the extent of LGE was higher in SLE patients with LN than in SLE patients without LN, the difference was not statistically significant (P > 0.05) (**Table 5**). In addition, there were no significant differences in GRS, GCS and GLS between SLE patients with LGE and those without LGE (P > 0.05). However, regardless of the subgroup of SLE patients with or without LGE, native T1, ECV, and T2 were higher in patients with LN, intermediate in patients without LN, and lower in the control group (P < 0.05).

**Table 5:** Impact of LGE on LV myocardium in patients with SLE.

Variables	Healthy controls (n=20)	SLE patients with LGE			SLE patients without LGE		
		Without LN (n=19)	With LN (n=11)	P*	Without LN (n=47)	With LN (n=25)	P*
LV strain							
GRS	40.01 ± 12.65	37.13 ± 14.29	31.57 ± 9.67	0.259	35.12 ± 9.81	36.48 ± 8.65	0.306
GCS	-18.88 ± 2.56	-19.42 ± 3.17	-19.25 ± 4.00	0.867	-19.49 ± 2.65	-19.28 ± 2.63	0.684
GLS	-14.92 ± 3.04	-14.11 ± 2.65	-10.09 ± 8.41	0.093	-14.51 ± 2.88	-13.36 ± 2.50	0.168
LV LGE							
LGE extent	-	3.62 ± 2.46	7.90 ± 6.47	0.101	-	-	-
LV tissue characterization							
Native T1	1256 ± 41	1324 ± 66†	1334 ± 63†	**<0.001**	1270 ± 82	1328 ± 52†‡	**<0.001**
ECV	26.20 ± 3.03	31.05 ± 2.06†	33.91 ± 5.99†	**<0.001**	27.36 ± 3.49	29.04 ± 2.82†	**0.013**
Native T2	39.60 ± 2.94	43.06 ± 2.98†	44.18 ± 5.07†	**0.001**	41.57 ± 3.84	43.48 ± 4.05†	**0.003**

Values are mean ± SD, number (%), or median (25th-75th percentile). Numbers in boldface indicate P values <0.05.

*P values are for overall comparisons across the three groups.

†P < 0.05 for pairwise comparisons versus the control group.

‡ P < 0.05 for pairwise comparisons between the SLE with LN group and the SLE without LN group.

LGE, late gadolinium enhancement; LV, left ventricular; SLE, systemic lupus erythematosus; LN, lupus nephritis; GRS, global radial strain; GCS, global circumferential strain; GLS, global longitudinal strain; ECV, extracellular volume.

### Impact of hypertension on the myocardium

In the SLE group, 24 patients (23.53%) had hypertension. To disentangle the effects of LN from hypertension, we performed hypertension-stratified analyses of mapping parameters and LGE. Compared with SLE patients without LN, SLE patients with LN tended to have a higher degree of LGE, but the difference was not statistically significant (P > 0.05). Similarly, there were no significant differences in GRS, GCS, and GLS across the groups (P > 0.05). Notably, in the SLE with or without hypertension subgroup, there was a stepwise increase in native T1, ECV, and T2 values respectively (P < 0.05) (**Table 4**). When stratified by hypertension status, native T1, ECV, and T2 values were numerically higher in LN patients in both strata (**Table 4**). In multivariable regression analyses, hypertension was independently associated with a greater LGE extent (β = 0.265, P < 0.05) (**Table 3**).

## Discussion

The present study showed that SLE patients with LN were associated with worsened clinical hematological indicators and a higher prevalence of hypertension. SLE patients with LN had higher native T1, ECV, and T2 values, despite preserved LVEF. These CMR findings indicate that LN could be associated with more severe diffuse myocardial involvement. The elevated native T1 and ECV are possible surrogates for interstitial expansion, which may include fibrosis, edema, or inflammation, while the elevated T2 more specifically suggests myocardial edema. Furthermore, hypertension was associated with an increase in the extent of LGE in SLE.

LN is a common manifestation in SLE patients, with an incidence rate of 35% in the present study. However, previous meta-analysis demonstrated that LN is associated with increased cardiovascular risk and mortality in patients with SLE (13). In line with previous studies, our study confirms that SLE patients with LN have a higher incidence rate of hypertension than patients without LN (2, 14). Laboratory profiles in the LN group were consistent with greater systemic and renal involvement, including higher inflammatory/immune markers and impaired renal indices, together with lower hemoglobin/hematocrit, albumin, and complement levels, which provide clinical context for the observed CMR differences.

In our cohort of SLE patients with LN, immune-inflammatory markers including neutrophils and erythrocyte sedimentation were elevated. Our results were consistent with previous findings that suggest SLE patients with LN are more likely to have elevated immune-inflammatory markers, which have been linked to atherosclerosis (13, 15). In addition, SLE patients with LN had lower hemoglobin, serum C4, and hematocrit than SLE patients without LN, which was negatively correlated with disease activity (16). Significantly, lactate dehydrogenase was elevated in SLE patients with LN compared with those without LN, although the mean values remained within the normal range. Collectively, these findings suggest that SLE patients with LN exhibit exacerbated immune dysregulation and a heightened systemic inflammatory response compared to those without LN (17). Furthermore, the relatively high prevalence of ANCA positivity in patients with LN may reflect overlapping immune dysregulation or underlying subclinical vasculitic features. This observation warrants further investigation into its potential clinical relevance.

LV strain derived from CMR feature tracking was considered as a promising tool that may be more sensitive than LVEF for detecting subtle LV systolic dysfunction (18). In the present study, SLE patients with LN had less negative GLS than those without LN, although GRS and GCS were normal. Because this difference was of borderline statistical significance, it should be interpreted cautiously as a subtle alteration in longitudinal deformation. Previous studies have shown that SLE patients with LN have more severe GLS impairment than patients with extra-renal SLE (6, 19). However, GLS in previous studies have usually been derived from echocardiography. Our study is the first to use LV strain derived from CMR feature tracking to assess the changes in myocardial mechanics in SLE patients with LN. In addition, our findings are consistent with prior ultrasound-based studies suggesting that GLS may detect subclinical dysfunction when LVEF is preserved (20, 21). Importantly, this subtle GLS difference occurred alongside more pronounced diffuse mapping abnormalities in LN patients, supporting the concept of subclinical myocardial involvement beyond conventional functional measures.

Notably, previous studies demonstrated that GLS is related to myocardial microvascular dysfunction, fat replacement, and fibrosis (22–24). Our study showed that SLE patients with LN had higher ECV, native T1, and were associated with a higher prevalence of hypertension compared with those without LN, although their LGE and the extent of LGE were comparable. Our findings suggest diffuse myocardial involvement in SLE, and mapping abnormalities may be more pronounced in patients with LN. These observations are consistent with the higher cardiovascular risk that has been reported in LN populations (5, 7, 13, 25). Further subgroup analysis showed that SLE patients with LN had a higher incidence rate of hypertension, which may be associated with additional myocardial remodeling (26). Moreover, multivariable analysis showed that the presence of hypertension was primarily linked to LGE extent, whereas LN status was more closely related to diffuse mapping abnormalities., which is consistent with previous findings (27). A clinically relevant observation is a differential pattern between diffuse and focal myocardial changes: LN status was primarily associated with diffuse mapping abnormalities (native T1/ECV/T2), which persisted irrespective of hypertension status, whereas hypertension was more closely related to focal myocardial injury as reflected by greater LGE extent. Notably, within the LGE-negative subgroup, LN persists as a significant determinant of elevated ECV and native T1 values in SLE patients. The greater elevations in ECV and native T1 in LN versus non-LN patients may reflect more pronounced diffuse myocardial involvement in LN.

Further analysis revealed that both native T1 and ECV were abnormally increased in LGE-negative SLE patients regardless of LN status. This finding demonstrates that a subset of SLE patients lacking conventional LGE evidence of focal fibrosis already exhibits diffuse myocardial abnormalities on mapping, which may precede detectable focal LGE. Prior research predominantly focused on SLE cohorts with detectable LGE, whereas our data emphasize the clinical relevance of subclinical myocardial remodeling in LGE-negative individuals. These results suggest that further research is needed to clarify whether refined risk stratification and management strategies could be beneficial in SLE (7, 28–30). On the other hand, we performed a subgroup analysis of SLE with and without hypertension to explore the potential confounding effect of hypertension. Of note, our study found that native T1 and ECV were also elevated in the group of SLE patients without hypertension, which means that LN may be associated with more pronounced diffuse myocardial involvement in SLE patients without hypertension.

In the pathophysiology of cardiac disease in SLE, immune complexes leading to myocardial oedema play a very important role in the mechanism of myocardial injury (31, 32). T2 mapping-CMR has been recommended as a non-invasive technique that can sensitively detect myocardial edema in the early subclinical myocardial involvement in SLE (5). Our study found that SLE patients with LN had elevated T2 values, indicating more severe myocardial edema in these patients. Previous studies showed that even in SLE patients with inactive disease, myocardial edema can be detected by T2 mapping-CMR (33, 34). Importantly, the SLE subjects with LN had more edema on T2 mapping-CMR and tended to have a greater extent of LGE. Given that SLE patients tend to be younger, intensive therapy to prevent LN complications may play a critical role in reducing long-term adverse cardiovascular events.

The prevalence of heart disease in SLE were high and usually without obvious cardiovascular symptoms, although the second peak of death which caused by cardiovascular disease in SLE patients (35, 36). In the present study, we confirmed that SLE patients with LN have more pronounced diffuse myocardial abnormalities on mapping (including higher T2 values consistent with edema and higher T1/ECV indicating interstitial expansion), which were more pronounced than those without LN, regardless of the presence of LGE or hypertension. In addition, GLS is reduced in patients with SLE and LN, which may provide additional information for myocardial mechanical injury. This may highlight that CMR may help with early detection of subclinical myocardial involvement, including myocardial fibrosis, edema, and systolic dysfunction in SLE patients with LN (5, 37). In addition, the cardiac damage in SLE patients with LN should be considered. Further prospective research with longitudinal follow-up is needed to determine the prognostic value of these multiparametric CMR indicators and to clarify their relationship with future cardiovascular outcomes in SLE, particularly in patients with LN.

### Limitations

This study had several limitations. First, it was a single-center, cross-sectional study with no follow-up, and therefore the associations observed cannot reliably establish causality. Second, a previous study found that the RV functional, rather than the LV, was involved; our study did not evaluate the histological parameters of the right ventricle, such as T1, T2 mapping, and strain (28). Because the thinner right ventricular wall may interfere with objective observation. Third, the number of LGE-positive patients was relatively small, and LGE extent is a semi-continuous, zero-inflated measure (with many participants showing no detectable LGE), which may limit statistical power and warrants cautious interpretation of analyses involving LGE extent. Fourth, residual confounding cannot be excluded in this retrospective cross-sectional study, particularly from immunosuppressive therapy and other medication exposures, renal dysfunction, and disease activity, which were not fully captured. Moreover, not all LN cases were biopsy-confirmed, and renal pathology details (class/activity/chronicity) and longitudinal characterization of renal activity were not uniformly available, which may introduce heterogeneity in LN phenotyping. Finally, our study did not conduct histopathological validation, and the pathological mechanism of severe myocardial injury in SLE patients with LN is currently unclear, and animal experiments are needed to verify this in the future.

## Conclusion

In conclusion, LN status in SLE was associated with a higher prevalence of hypertension and a pattern of diffuse myocardial involvement on CMR (higher native T1/ECV and T2) with a modest reduction in GLS. Future prospective studies are warranted to determine the prognostic significance of these CMR abnormalities and to evaluate the potential value of structured cardiac assessment strategies in this population.

## Data Availability

The raw data supporting the conclusions of this article will be made available by the authors, without undue reservation.

## Supplementary material

Supplementary Figure 1 Flow diagram of participant selection and group allocation.

## References

[1] Tzeng HT Chyuan IT . Immunometabolism in systemic lupus erythematosus: Relevant pathogenetic mechanisms and potential clinical applications. J Formos Med Assoc. (2021) 120:1667–75. doi: 10.1016/j.jfma.2021.03.019, PMID: 33836940

[2] Hasan MA Alali L Alsadah F Alobud S Alsaif J Alali Z . Prevalence and patterns of renal involvement among patients with systemic lupus erythematous at a tertiary center. J Clin Rheumatol. (2023) 29:84–90. doi: 10.1097/RHU.0000000000001914, PMID: 36251502

[3] Lu X Wang Y Zhang J Pu D Hu N Luo J . Patients with systemic lupus erythematosus face a high risk of cardiovascular disease: A systematic review and Meta-analysis. Int Immunopharmacol. (2021) 94:107466. doi: 10.1016/j.intimp.2021.107466, PMID: 33636561

[4] Symmons DP Gabriel SE . Epidemiology of CVD in rheumatic disease, with a focus on RA and SLE. Nat Rev Rheumatol. (2011) 7:399–408. doi: 10.1038/nrrheum.2011.75, PMID: 21629241

[5] Luo S Dou WQ Schoepf UJ Varga-Szemes A Pridgen WT Zhang LJ . Cardiovascular magnetic resonance imaging in myocardial involvement of systemic lupus erythematosus. Trends Cardiovasc Med. (2023) 33:346–54. doi: 10.1016/j.tcm.2022.02.002, PMID: 35150849

[6] Zhong XF Chen LX Liu LX Peng GJ Luo SY Liu DS . Early detect left ventricular subclinical myocardial dysfunction in patients with systemic lupus erythematosus by a left ventricular pressure-strain loop. Lupus. (2022) 31:596–605. doi: 10.1177/09612033221089150, PMID: 35348025

[7] Puntmann VO D’Cruz D Smith Z Pastor A Choong P Voigt T . Native myocardial T1 mapping by cardiovascular magnetic resonance imaging in subclinical cardiomyopathy in patients with systemic lupus erythematosus. Circ Cardiovasc Imaging. (2013) 6:295–301. doi: 10.1161/CIRCIMAGING.112.000151, PMID: 23403334

[8] Winau L Hinojar Baydes R Braner A Drott U Burkhardt H Sangle S . High-sensitive troponin is associated with subclinical imaging biosignature of inflammatory cardiovascular involvement in systemic lupus erythematosus. Ann Rheum Dis. (2018) 77:1590–8. doi: 10.1136/annrheumdis-2018-213661, PMID: 30077990

[9] du Toit R Herbst PG Ackerman C Pecoraro AJ Claassen D Cyster HP . Outcome of clinical and subclinical myocardial injury in systemic lupus erythematosus - A prospective cohort study. Lupus. (2021) 30:256–68. doi: 10.1177/0961203320976960, PMID: 33525979

[10] Aringer M Costenbader K Daikh D Brinks R Mosca M Ramsey-Goldman R . 2019 European league against rheumatism/American college of rheumatology classification criteria for systemic lupus erythematosus. Arthritis Rheumatol. (2019) 71:1400–12. doi: 10.1002/art.40930, PMID: 31385462 PMC6827566

[11] Patrizi ST Tandel MD Boothroyd D Simard JF Hsu JJ . Prognostic value of the 2019 EULAR/American college of rheumatology systemic lupus erythematosus classification criteria to renal response one year after treatment in a cohort with childhood-onset lupus nephritis. ACR Open Rheumatol. (2024) 6:454–62. doi: 10.1002/acr2.11674, PMID: 38695166 PMC11319914

[12] Siegel CH Sammaritano LR . Systemic lupus erythematosus: A review. JAMA. (2024) 331:1480–91. doi: 10.1001/jama.2024.2315, PMID: 38587826

[13] Wong CY Ma BMY Zhang D Cheung W Chan TM Yap DYH . Cardiovascular risk factors and complications in patients with systemic lupus erythematosus with and without nephritis: a systematic review and meta-analysis. Lupus Sci Med. (2024) 11:e001152. doi: 10.1136/lupus-2024-001152, PMID: 38519060 PMC10961538

[14] Saavedra M Gracia-Aréchiga TS Miranda-Hernández D Sánchez A Arrucha-Cozaya M Cruz-Domínguez MDP . Active but not quiescent lupus nephritis during pregnancy is associated with a higher rate of adverse obstetric outcomes: Analysis of a prospective cohort. Int J Gynaecol Obstet. (2024) 167:420–6. doi: 10.1002/ijgo.15601, PMID: 38736284

[15] Rabrenović V Petrović M Rabrenović M Pilčević D Rančić N . The significance of biomarkers of inflammation in predicting the activity of Lupus nephritis. J Med Biochem. (2024) 43:116–25. doi: 10.5937/jomb0-43457, PMID: 38496018 PMC10943464

[16] Wang H Lan L Chen J Xiao L Han F . Peripheral blood T-cell subset and its clinical significance in lupus nephritis patients. Lupus Sci Med. (2022) 9:e000717. doi: 10.1136/lupus-2022-000717, PMID: 35973743 PMC9386235

[17] Song K Liu X Liu J Yin Z Chen P Cai G . Analysis of clinical and laboratory characteristics and pathology of lupus nephritis-based on 710 renal biopsies in China. Clin Rheumatol. (2020) 39:3353–63. doi: 10.1007/s10067-020-05115-2, PMID: 32435895

[18] Yang W Li H He J Yin G An J Forman C . Left ventricular strain measurements derived from MR feature tracking: A head-to-head comparison of a higher temporal resolution method with a conventional method. J Magn Reson Imaging. (2022) 56:801–11. doi: 10.1002/jmri.28053, PMID: 35005810

[19] He W Xie P Li W Yao F Liu Y Liang L . Impaired left ventricular systolic synchrony in patients with lupus Nephritis: A speckle tracking echocardiography study. Lupus. (2022) 31:1084–93. doi: 10.1177/09612033221102713, PMID: 35575173

[20] Gegenava M Kirtava Z Kong WK Gegenava T . Left ventricular systolic function assessed by standard and advanced echocardiographic techniques in patients with systemic lupus erythematosus: A systemic review and meta-analysis. Arch Rheumatol. (2024) 39:149–58. doi: 10.46497/ArchRheumatol.2024.10131, PMID: 38774698 PMC11104758

[21] Li X Chen H Han M Luo Y Liu F Chen L . Quantitative assessment of left ventricular systolic function in patients with systemic lupus erythematosus: a non-invasive pressure-strain loop technique. Quant Imaging Med Surg. (2022) 12:3170–83. doi: 10.21037/qims-21-951, PMID: 35655829 PMC9131320

[22] Grimault D Serfaty JM Guyomarch B Marteau L Goudal A Schmitt S . Description of the two dimensional layer-specific strain echocardiography phenotype of arrhythmogenic left ventricular cardiomyopathy. J Am Soc Echocardiogr. (2024) 37:960–70. doi: 10.1016/j.echo.2024.05.017, PMID: 38823601

[23] Bojer AS Sørensen MH Madsen SH Broadbent DA Plein S Gæde P . Early signs of myocardial systolic dysfunction in patients with type 2 diabetes are strongly associated with myocardial microvascular dysfunction independent of myocardial fibrosis: a prospective cohort study. Diabetol Metab Syndr. (2024) 16:41. doi: 10.1186/s13098-024-01285-0, PMID: 38350975 PMC10863286

[24] Haland TF Almaas VM Hasselberg NE Saberniak J Leren IS Hopp E . Strain echocardiography is related to fibrosis and ventricular arrhythmias in hypertrophic cardiomyopathy. Eur Heart J Cardiovasc Imaging. (2016) 17:613–21. doi: 10.1093/ehjci/jew005, PMID: 26873460 PMC4871235

[25] Wu R An DA Hu J Jiang M Guo Q Xu JR . The apparent diffusion coefficient is strongly correlated with extracellular volume, a measure of myocardial fibrosis, and subclinical cardiomyopathy in patients with systemic lupus erythematosus. Acta Radiol. (2018) 59:287–95. doi: 10.1177/0284185117717763, PMID: 28679323

[26] Kwiecinski J Lennen RJ Gray GA Borthwick G Boswell L Baker AH . Progression and regression of left ventricular hypertrophy and myocardial fibrosis in a mouse model of hypertension and concomitant cardiomyopathy. J Cardiovasc Magn Reson. (2020) 22:57. doi: 10.1186/s12968-020-00655-7, PMID: 32758255 PMC7409657

[27] Jiang L Ren Y Yu H Guo YK Liu X Deng MY . Additive effect of hypertension on left ventricular structure and function in patients with asymptomatic type 2 diabetes mellitus. J Hypertens. (2021) 39:538–47. doi: 10.1097/HJH.0000000000002643, PMID: 33031176

[28] Guo Q Wu LM Wang Z Shen JY Su X Wang CQ . Early detection of silent myocardial impairment in drug-naive patients with new-onset systemic lupus erythematosus: A three-center prospective study. Arthritis Rheumatol. (2018) 70:2014–24. doi: 10.1002/art.40671, PMID: 30070061

[29] Cui B Pu H Liu J He W Zhou Y Zhou X . The effect of community-acquired pneumonia on myocardial injury in systemic lupus erythematosus: Insights from cardiac magnetic resonance. Lupus. (2022) 31:1263–8. doi: 10.1177/09612033221106304, PMID: 35667652

[30] Mavrogeni S Bratis K Markussis V Spargias C Papadopoulou E Papamentzelopoulos S . The diagnostic role of cardiac magnetic resonance imaging in detecting myocardial inflammation in systemic lupus erythematosus. Differentiation from viral myocarditis. Lupus. (2013) 22:34–43. doi: 10.1177/0961203312462265, PMID: 23035042

[31] Sierra-Galan LM Bhatia M Alberto-Delgado AL Madrazo-Shiordia J Salcido C Santoyo B . Cardiac magnetic resonance in rheumatology to detect cardiac involvement since early and pre-clinical stages of the autoimmune diseases: A narrative review. Front Cardiovasc Med. (2022) 9:870200. doi: 10.3389/fcvm.2022.870200, PMID: 35911548 PMC9326004

[32] Greulich S Ferreira VM Dall’Armellina E Mahrholdt H . Myocardial inflammation-are we there yet? Curr Cardiovasc Imaging Rep. (2015) 8:6. doi: 10.1007/s12410-015-9320-6, PMID: 25705323 PMC4330458

[33] Shalmon T Thavendiranathan P Seidman MA Wald RM Karur GR Harvey PJ . Cardiac magnetic resonance imaging T1 and T2 mapping in systemic lupus erythematosus in relation to antimalarial treatment. J Thorac Imaging. (2023) 38:W33–w42. doi: 10.1097/RTI.0000000000000703, PMID: 36917505

[34] Zhang Y Corona-Villalobos CP Kiani AN Eng J Kamel IR Zimmerman SL . Myocardial T2 mapping by cardiovascular magnetic resonance reveals subclinical myocardial inflammation in patients with systemic lupus erythematosus. Int J Cardiovasc Imaging. (2015) 31:389–97. doi: 10.1007/s10554-014-0560-3, PMID: 25352245

[35] Urowitz MB Bookman AA Koehler BE Gordon DA Smythe HA Ogryzlo MA . The bimodal mortality pattern of systemic lupus erythematosus. Am J Med. (1976) 60:221–5. doi: 10.1016/0002-9343(76)90431-9, PMID: 1251849

[36] Faurschou M Mellemkjaer L Starklint H Kamper AL Tarp U Voss A . High risk of ischemic heart disease in patients with lupus nephritis. J Rheumatol. (2011) 38:2400–5. doi: 10.3899/jrheum.110329, PMID: 21885497

[37] Khayata M Wang TKM Chan N Alkharabsheh S Verma BR Oliveira GH . Multimodality cardiac imaging in patients with systemic lupus erythematosus. Curr Probl Cardiol. (2023) 48:101048. doi: 10.1016/j.cpcardiol.2021.101048, PMID: 34774920

