# Supplementary figures and images for: Lupus nephritis and its association with subclinical myocardial alterations in systemic lupus erythematosus assessed by cardiovascular magnetic resonance
Source: Front Immunol. 2026 Feb 6;17:1749478. doi: 10.3389/fimmu.2026.1749478 (PMC12920543; doi:10.3389/fimmu.2026.1749478)

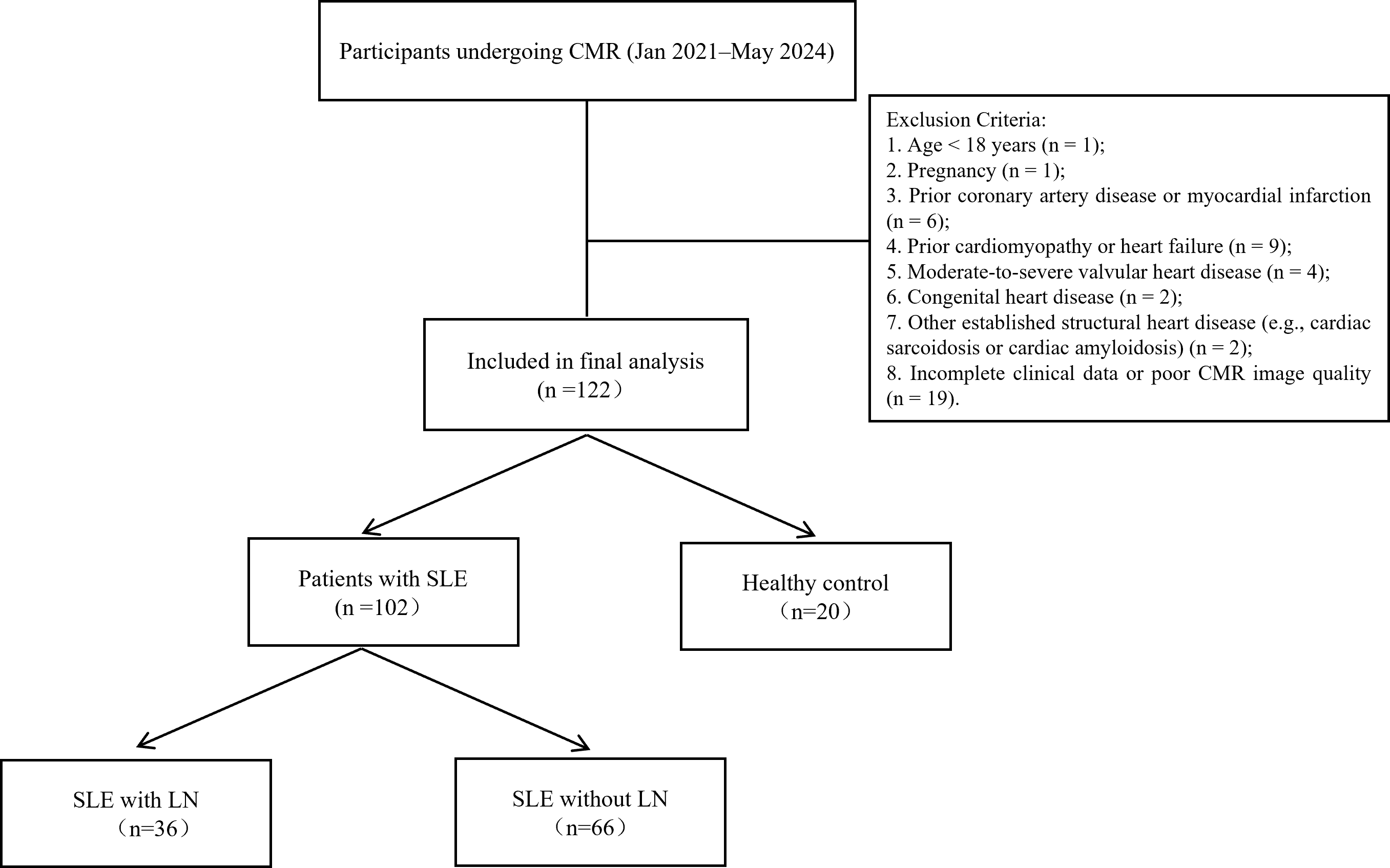


**Supplementary Figure 1** Flow diagram of participant selection and group allocation

Supplement: Supplementary Figure 1 — Flow diagram of participant selection and group allocation. [file Table1.docx]
